# Sex‐dependent influence of postweaning environmental enrichment in Angelman syndrome model mice

**DOI:** 10.1002/brb3.2468

**Published:** 2022-01-04

**Authors:** Jameson A. Cosgrove, Lauren K. Kelly, Elizabeth A. Kiffmeyer, Alexander D. Kloth

**Affiliations:** ^1^ Department of Biology Augustana University 2001 S. Summit Avenue Sioux Falls South Dakota USA

**Keywords:** behavior therapy, mouse, neurodevelopmental disorders, sex differences, transgenic

## Abstract

**Introduction:**

Angelman syndrome (AS) is a rare neurodevelopmental disorder caused by mutation or loss of *UBE3A* and marked by intellectual disability, ataxia, autism‐like symptoms, and other atypical behaviors. One route to treatment may lie in the role that environment plays early in postnatal life. Environmental enrichment (EE) is one manipulation that has shown therapeutic potential in preclinical models of many brain disorders, including neurodevelopmental disorders. Here, we examined whether postweaning EE can rescue behavioral phenotypes in *Ube3a* maternal deletion mice (AS mice), and whether any improvements are sex‐dependent.

**Methods:**

Male and female mice (C57BL/6J *Ube3a^tm1Alb^* mice and wild‐type (WT) littermates; ≥10 mice/group) were randomly assigned to standard housing (SH) or EE at weaning. EE had a larger footprint, a running wheel, and a variety of toys that promoted foraging, burrowing, and climbing. Following 6 weeks of EE, animals were submitted to a battery of tests that reliably elicit behavioral deficits in AS mice, including rotarod, open field, marble burying, and forced swim; weights were also monitored.

**Results:**

In male AS‐EE mice, we found complete restoration of motor coordination, marble burying, and forced swim behavior to the level of WT‐SH mice. We also observed a complete normalization of exploratory distance traveled in the open field, but we found no rescue of vertical behavior or center time. AS‐EE mice also had weights comparable to WT‐SH mice. Intriguingly, in the female AS‐EE mice, we found a failure of EE to rescue the same behavioral deficits relative to female WT‐SH mice.

**Conclusions:**

Environmental enrichment is an effective route to correcting the most penetrant phenotypes in male AS mice but not female AS mice. This finding has important implications for the translatability of early behavioral intervention for AS patients, most importantly the potential dependency of treatment response on sex.

## Graphical abstract

Environmental enrichment (EE) is a powerful behavioral manipulation that may provide a route to ameliorating the symptoms of some brain disorders. In this application of EE in mice modeling Angelman syndrome (AS), a rare neurodevelopmental disorder, we found that this intervention can correct a wide range of robust behavioral deficits commonly seen in male AS mice. Notably, we also found that female AS mice do respond to the same intervention, which constitutes a key sex difference that will need to be considered by researchers and therapists alike in the future.

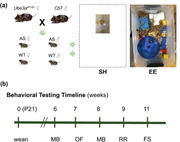

## 1 INTRODUCTION

Angelman syndrome (AS) is a neurodevelopmental disorder that affects at least one in 20,000 individuals worldwide (Buiting et al., 2016; Dan, 2009; Mabb et al., 2011; Margolis et al., 2015), with equal numbers of males and females affected by the disorder (Buiting et al., 2016). AS is marked by developmental delay, absent or impaired speech, severe intellectual disability, sleep disruption, and a series of other behavioral abnormalities including motor dysfunction, hyperactivity, and anxiety (Margolis et al., 2015; Williams et al., 2006). Neurologically, AS patients have microcephaly, abnormal brain rhythmicity, and high seizure susceptibility (Dan, 2009; Sidorov et al., 2017). A large fraction of AS patients also has a comorbid diagnosis of autism spectrum disorder (Veltman et al., 2005). Moreover, like other neurodevelopmental disorders, AS constitutes a tremendous lifelong hardship for patients, their families, and the healthcare system (Wheeler et al., 2017). For this reason, it is imperative to develop therapeutic approaches that effectively treat the core features of AS.

Preclinical AS research has made extensive use of transgenic mouse models with disruptions of the gene *Ube3a*, which encodes an E3 ubiquitin ligase that is critical for brain maturation and synaptic communication (Mabb et al., 2011). The most frequently used mouse model, originally generated by Jiang and colleagues in 1998, has a behavioral profile that echoes the clinical presentation in AS patients (Jiang et al., 1998; Sonzogni et al., 2018). These mice show robust and highly reproducible deficits in a battery of behavioral tasks that includes the accelerating rotarod, which tests motor coordination and motor learning; the open‐field task, which tests activity and anxiety; and other tasks including the marble‐burying task, the nest building task, and the forced swim task (Allensworth et al., 2011; Born et al., 2017; Huang et al., 2013; Jiang et al., 1998; Sonzogni et al., 2018). Some studies have also demonstrated high seizure susceptibility, disrupted brain rhythmicity, and mild cognitive impairments (Born et al., 2017; Dan, 2009; Huang et al., 2013; Jiang et al., 1998; Sidorov et al., 2017; Sonzogni et al., 2018), as well as sex‐dependent sensory defects (Koyavski et al., 2019) in these animals. Work with AS mouse models has revealed a series of promising pharmacological (Baudry et al., 2012; Ciarlone et al., 2017; Cruz et al., 2021; Gu et al., 2019; Guzzetti et al., 2018; Hethorn et al., 2015; Huang et al., 2013; Liu et al., 2019; van Woerden et al., 2007), dietary (Ciarlone et al., 2017), and gene‐therapy (Meng et al., 2013; Schmid et al., 2021; Silva‐Santos et al., 2015; Sonzogni et al., 2019; Wolter et al., 2020) approaches that counteract the disruption of *Ube3a* and correct these behavioral phenotypes. Despite these efforts, the only approved clinical therapies to date are those that treat symptoms such as seizure onset and sleep loss (Bi et al., 2016; Markati et al., 2021; Rotaru et al., 2020).

Recently, there has been interest in understanding the role that early‐life experience plays in neurodevelopmental disorders and whether modification of the early‐life environment would constitute an effective route to treatment (Consorti et al., 2019; Hannan, 2014; Kelly & Hannan, 2019; Kondo & Hannan, 2019; Sale et al., 2014). Using environmental enrichment (EE)—an experimental paradigm in which researchers provide additional sensory, cognitive, social, or physical stimulation to their subjects (Nithianantharajah & Hannan, 2006; Sztainberg & Chen, 2010)—researchers have been able to correct phenotypes in mouse models of a variety of neuropsychiatric disorders. For instance, in juvenile mice modeling Rett syndrome, a disorder that shares several features with AS, cognitive and physical enrichment was able to restore motor function on the rotarod (Kondo et al., 2008; Nag et al., 2009), and earlier intervention was able to restore spatial learning (Lonetti et al., 2010). This approach has successfully rescued alterations in activity, motor function, repetitive behaviors, and other autism‐related behaviors in other mouse models of neurodevelopmental disorders (Lacaria et al., 2012; Martínez‐Cué et al., 2002; Queen et al., 2020; Restivo et al., 2005; Reynolds et al., 2013; Schneider et al., 2006; Yamaguchi et al., 2017). Promisingly, experts in autism therapy have been able to translate some aspects of EE into clinical therapies and have found some success with their patients (Woo & Leon, 2013; Woo et al., 2015). In AS mice, there is evidence that altering the sensory environment in adult animals is sufficient to rescue synaptic plasticity in the visual cortex, suggesting that other disease‐related phenotypes may also be susceptible to change by environmental enrichment (Yashiro et al., 2009). Adding to the potential of EE for AS mice, one study that applied long‐term EE to male mice showed improvement in a small number of behaviors (Jamal et al., 2017). However, it remains unclear how EE affects the most robust and highly reproducible behavior phenotypes of AS in mice, and it is important to explore how sex, enrichment time, and other parameters determine the effectiveness of EE as an intervention.

In the present study, we investigated the degree to which 6–12 weeks postweaning EE rescues behavioral phenotypes in AS model mice. We examined a battery of behaviors that has proven to be highly reproducible across investigators and AS mouse models (Sonzogni et al., 2018) and is broader in scope than previous studies. At the same time, we asked whether the rescue of any phenotype due to EE was dependent on sex, as sex differences are important to document when evaluating the effectiveness of any potential treatment (Bale & Epperson, 2017; Shansky & Woolley, 2016). We found that while this form of EE is capable of completely or partially rescuing many highly penetrant behaviors in male mice, it is considerably less effective in female mice. The substantial sex difference demonstrated here has important implications for behavioral therapeutic approaches to AS.

## 2 MATERIALS AND METHODS

### 2.1 Animals

**FIGURE 1:**
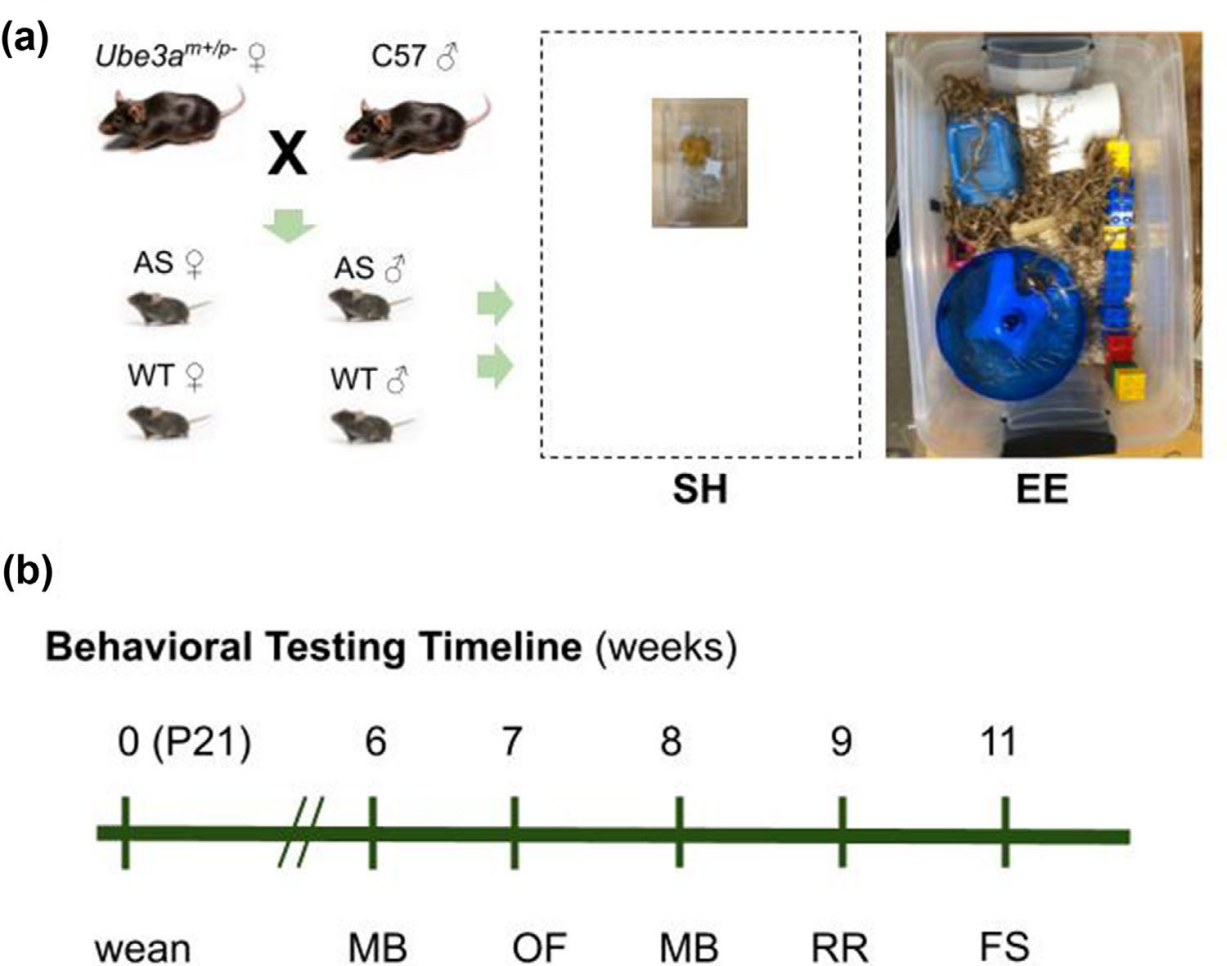
Experimental setup. (a) Angelman syndrome (AS, *Ube3a^m‐/p+^)* mice on the C57BL/6J (C57) background and their wild‐type (WT) littermates arising from mothers carrying the paternal deletion (*Ube3a^m+/p–^)* and WT males (left) were randomly assigned to standard housing (SH) or larger enriched housing (EE) (right). Dashed line indicates the relative size of EE housing. (b) Timeline for behavioral testing. Following the beginning of enrichment at weaning at postnatal day 21, marble burying (MB), open field (OF), accelerating rotarod (RR), and forced swim (FS) tasks were administered

Male and female heterozygous mice carrying a maternal deletion of *Ube3a (Ube3a^m+/p–^*, denoted as AS) and wild‐type (WT) littermates were bred at Augustana University. Female heterozygous mice on the C57 background carrying a paternal deletion of *Ube3a* (*Ube3a^tm1Alb^*; stock No. 016590; RRID:JAX_IMSR:016590; Jiang et al., 1998) and male C57BL/6J mice (stock No. 000664; RRID:IMSR_JAX:000664) were obtained from Jackson Laboratories (Bar Harbor, ME, USA) and paired for breeding in standard housing (SH). Genotype was confirmed by PCR testing according to previously published protocols (Jiang et al., 1998; Judson et al., 2016). At postnatal day 21, pups were separated by sex and weaned randomly into SH or environmentally enriched housing; litters were not separated by genotype (Figure 1a). In total, 110 mice from 15 litters from four breeding pairs were used (WT‐SH, 17 males and 11 females; WT‐EE, 19 males and 16 females; AS‐SH, 13 males and 11 females; and AS‐EE, 13 males and 10 females). These numbers do not deviate significantly from the expected values of 25% male WT mice, 25% male AS mice, 25% female WT mice, and 25% female AS mice from this breeding scheme (chi‐squared test, *χ* ^2^(3) = 4.255, *p* = .23).

All mice were housed on a 12‐h light‐dark cycle (7 a.m.–7 p.m.) in open‐top filtered mouse cages (Ancare, Bellmore, NY, USA) in groups of 2–5 littermates per cage (median = 3 littermates, interquartile range = 1 littermate). Animals had ad libitum access to food and water during this period. Cages were changed on a biweekly basis. All experimental procedures complied with NIH guidelines and were approved by the Augustana University Institutional Animal Care and Use Committee.

### 2.2 Housing conditions

At weaning, mice were assigned randomly and blind to genotype to one of two housing conditions (Figure 1a) and housed exclusively with their littermates. Animals from no more than one litter were assigned to the same cage. SH consisted of a 19 cm × 29.2 cm × 12.7 cm conventional open‐top filtered mouse cage (Ancare) that contained aspen bedding (Envigo, Indianapolis, IN, USA), a shelter (Fisher Scientific, Waltham, MA, USA) and compressed cotton nesting material (nestlet, Ancare) along with a food hopper and a water bottle. Environmentally enrichment housing was modeled after prior literature on environmental enrichment in mice (Nithianantharajah & Hannan, 2006; Sztainberg & Chen, 2010; Tomas et al., 2015) and consisted of opportunities for physical exercise, such as running and climbing; for exploration of a variety of textures, materials, and sensory modalities; and for digging and burrowing. The cage consisted of a 46 cm × 31.1 cm × 17.8 cm arena made of clear plastic (Sterilite, Townsend, MA, USA) custom‐adapted to fit a filtered cage top, food hopper, and water bottle (Ancare). The enclosure contained aspen bedding (Envigo); a variety of shelter options, including red and yellow translucent houses and huts (Fisher Scientific), “walk‐up” houses (Petmate, Arlington, TX, USA), and upcycled plastic piping (purchased from the Scrap Exchange, Durham, NC, USA); one of two types of exercise wheel (Fisher Scientific, and Ware Pet Products, Phoenix, AZ, USA); compressed cotton nestlets; (nestlet, Fisher Scientific) crinkle paper (Crink‐l'Nest, Lab Supply, Dallas, TX, USA); treats (Hartz) scattered throughout the bedding; and an assortment of toys, including bells, Nylabones (Neptune City, NJ, USA), wooden blocks and sticks, chains, ropes, activity rings (Bio‐Serv, Flemington, NJ, USA) Duplo blocks (LEGO, Enfield, CT, USA), and balls of a variety of sizes and textures. Enrichment devices were changed out once a week, and the locations of enrichment devices and the orientation of the enrichment cage in the housing room were rotated once a week.

### 2.3 Behavioral testing battery

After 6 weeks in the assigned housing, mice underwent a series of behavioral assays over the next 5 weeks (Figure 1b). These behaviors were selected from among the “gold standard” of highly reproducible phenotypes in AS mice on the C57 background across laboratories (Rotaru et al., 2020). All mice underwent marble burying, rotarod, and open‐field testing. Forced swim testing, which began after 18 mice had already completed the behavioral battery, was administered to 92 mice. Testing occurred during light hours. Prior to each testing session, the mice were acclimated to the testing room for a minimum of 30 min. Following each behavioral assay, mice were returned to their home cages. Mice were weighed weekly during the behavioral battery. All data were collected by experimenters blind to genotype.

#### 2.3.1 Marble burying

Marble‐burying measures ethologically relevant repetitive behavior in mice (Angoa‐Pérez et al., 2013; Deacon, 2006). Mice were placed in a Plexiglas cage (Ancare) containing 5 cm of ⅛′′ corncob bedding (The Andersons Inc., Maumee, OH, USA) and 15 ¼′′ black glass marbles arranged in a 3‐by‐5 grid; testing took place in the presence of 60 dB white noise under dim light (30–40 lux). The mice were allowed to interact with the marbles for 30 min, after which the animals were removed from the cage and the number of unburied marbles was counted. A marble was counted as unburied if less than 50% of its surface was covered. The average number of unburied marbles was calculated for each cage from the independent observations of three researchers. The inside of the cage was cleaned with 70% ethanol between trials and bedding was replaced.

#### 2.3.2 Rotarod

Testing on the accelerating rotarod, which measures motor function and motor coordination (Deacon, 2013), was carried out as previously described (Gu et al., 2019; Huang et al., 2013; Thaxton et al., 2018). Briefly, mice were tested on 2 separate days, with three trials delivered on the first day and two trials delivered 48 h later. Each day began with 30 min of habituation in a brightly lit room. During each trial, mice were placed using a wooden dowel into one of four lanes of a rod rotating at a constant speed of 4 rpm. Once the trial began, the rotarod accelerated to a speed of 40 rpm over 5 min. The trial for each mouse ended when the mouse fell off the rotarod, completed two complete somersaults around the rotarod, or reached the end of the 5 min trial; end‐of‐trial times were recorded. Subsequent trials on the same day started 10 min later. The rotarod was cleaned with 70% ethanol between trials.

#### 2.3.3 Open field

The open‐field task measures anxiety and exploratory activity in mice (Sonzogni et al., 2018; Tanaka et al., 2012). Mice were placed in a 41 cm × 41 cm × 41 cm white Plexiglas testing chamber; testing took place under bright lights and in the presence of 60 dB white noise. The mice were allowed to freely roam the testing chamber for 30 min while being recorded by a PSEye camera (Sony Interactive Entertainment, San Mateo, CA, USA) mounted on the ceiling (Badura et al., 2018; Kloth et al., 2015), with images collected at a rate of 75 fps. After testing, the mice were removed from the testing chamber and returned to their home cages, and the inside of the testing chamber was cleaned with 70% ethanol. Video was recorded using a custom Python (RRID:SCR_008394) program using the CL‐Eye Platform (Code Laboratories, Henderson, NV, USA) (Badura et al., 2018; Kloth et al., 2015) and was analyzed using custom MATLAB (RRID:SCR_001622) code similar to previously published methods (Zhang et al., 2020). The MATLAB analysis extracted distance traveled and entries into the center (defined as at least 10.25 cm from the walls of the arena). Videos were also scored manually by experimenters blind to genotype and housing condition in order to quantify the number of rearing episodes.

#### 2.3.4 Forced swim

The forced swim task tests depressive‐like behavior in mice (Can et al., 2012). Following 30 min habituation under dim lights (30–40 lux), mice were placed in a clear, plastic 3 L beaker (Fisher Scientific) filled with water (approximately 25°C) and were allowed to acclimate for 2 min. After acclimation, the mice were allowed to swim for another 4 min while being recorded. After testing, the video was scored manually by experimenters blind to genotype and housing condition in order to determine the amount of time each mouse spent immobile and the amount of time each mouse spent producing rhythmic, propulsive movements of the hindlimbs and forelimbs.

### 2.4 Statistics

Data were analyzed using two‐way analyses of variance (ANOVAs) unless otherwise noted. Homoscedasticity of residuals was confirmed using Spearman's rank correlation test, and normality of residuals was confirmed using Shapiro–Wilk's test. In some cases, data were transformed to satisfy the assumptions of the two‐way ANOVA: marble‐burying data were transformed as $Y\;=\;\sqrt{Y}$, while other data were transformed as Y=log(Y). These cases are clearly indicated in Supporting Information. Significant genotype or housing effects were followed up with Bonferroni‐corrected planned comparisons between the following pairs of groups: WT‐SH versus WT‐EE, AS‐SH versus AS‐EE, WT‐SH versus AS‐SH, and AS‐EE versus WT‐SH. The data were analyzed using Graphpad Prism 9 (GraphPad Software, San Diego, CA; RRID:SCR_002798). The significance level was *α* = .05. All results depicted in figures are the mean ± SEM of the untransformed data. Complete statistical information can be found in Supporting Information.

## 3 RESULTS

Following 6 weeks of postweaning experimental housing, mice underwent behavioral testing (Figure 1). Complete results from statistical analyses can be found in Supporting Information.

### 3.1 Weights

**FIGURE 2:**
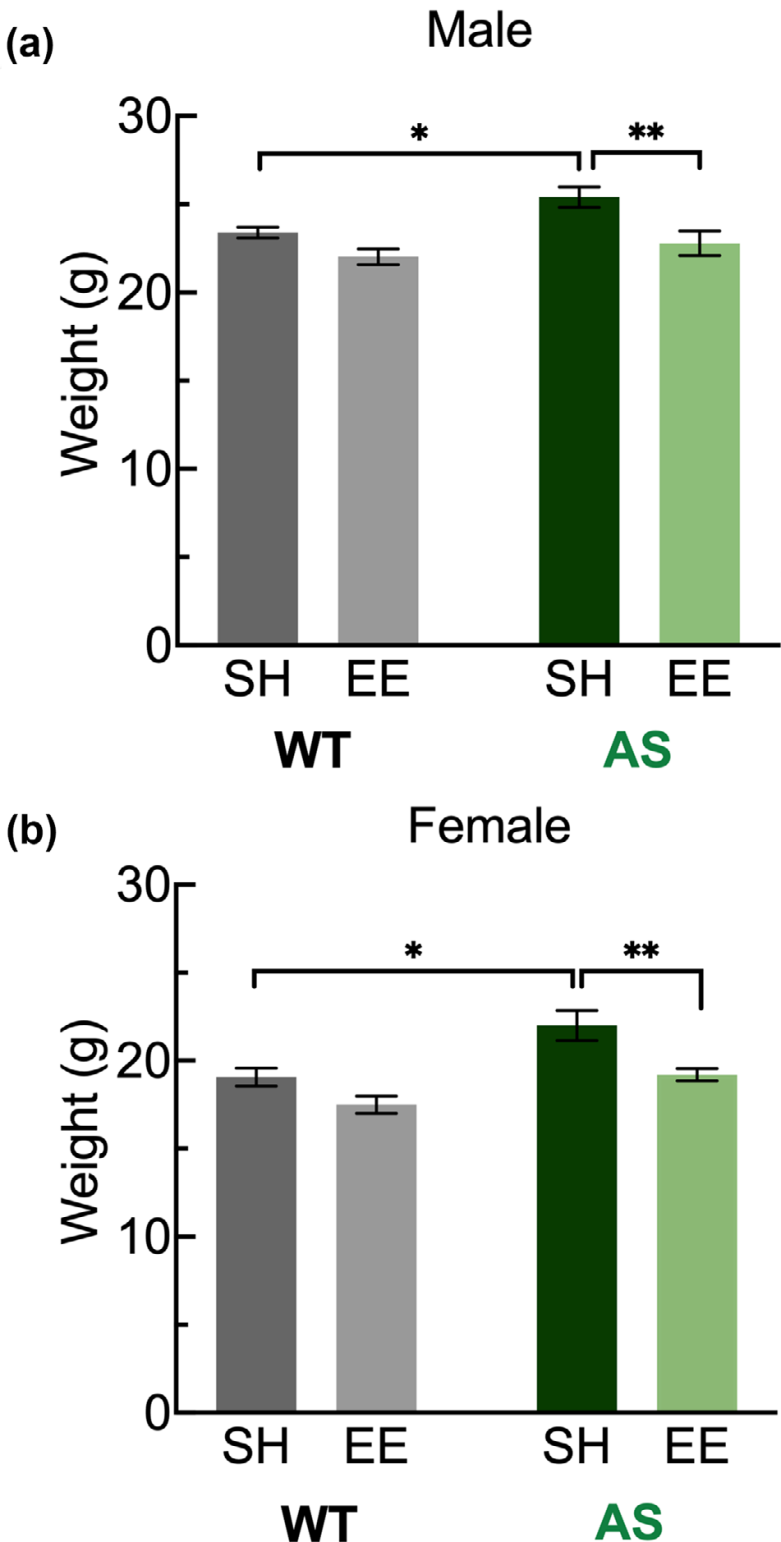
Environmental enrichment normalizes weight deficit at week 6. Improvements in excessive weights were observed in male (a) and female (b) AS mice in the enriched environment. Abbreviations: AS, Angelman syndrome mice; EE, enriched environment; SH, standard housing; WT, wild‐type littermates. Weights, in grams, displayed as mean ± SEM. Two‐way analysis of variance (ANOVA) followed by Bonferroni‐corrected planned comparisons: *p < .05, **p < .01

First, we tested the effect of housing at 6 weeks postweaning on the excess weight phenotype documented in AS mice (Huang et al., 2013). Two‐way ANOVA analysis of weights from male mice revealed significant main effects of both genotype (*p* = .0068) and housing (*p* = .0003) (Figure 2a). Increased weight seen in AS‐SH mice (vs. WT‐SH, *p* = .0121) was normalized by EE: AS‐EE weights were significantly different from AS‐SH weights (*p* = 0.0021) and could not be distinguished from WT‐SH animals (*p* > .9999). Likewise, two‐way ANOVA analysis of weights from female mice revealed significant main effects of both genotype (*p* = .0001) and housing (*p* = .0003) (Figure 2b). Increased weight seen in AS‐SH mice (vs. WT‐SH, *p* = .0297) was also completely normalized by EE: AS‐EE weights were significantly different from AS‐SH weights (*p* = .0034) and could not be distinguished from WT‐SH animals (*p* > .9999).

### 3.2 Marble burying

**FIGURE 3:**
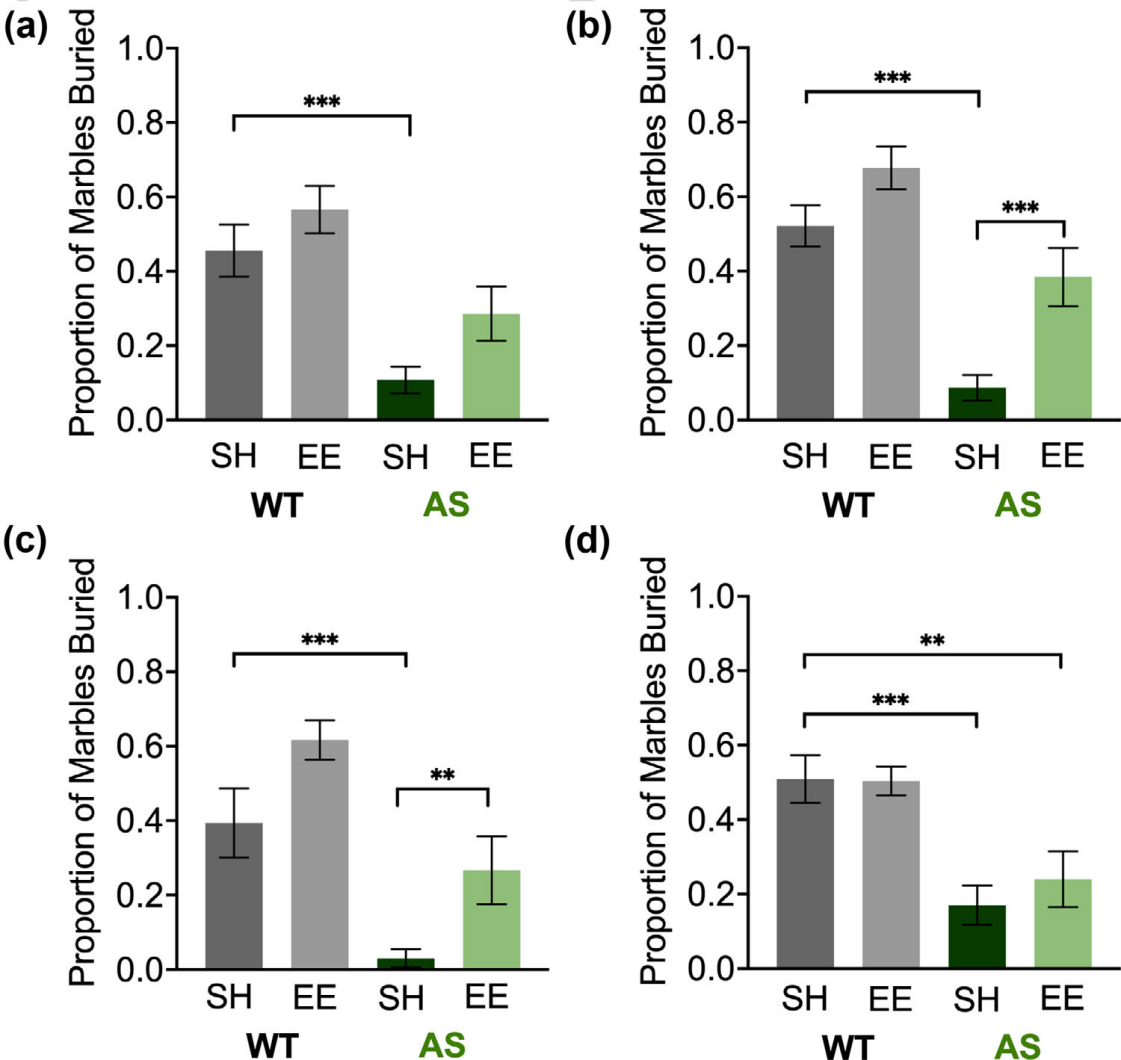
Environmental enrichment normalizes marble burying in male AS mice after 8 weeks. Improvement in the deficit in the number of marbles buried was observed at week 6 in male (a) and female (b) AS mice in the enriched environment, with this improvement strengthened in male AS mice (c) but not female AS mice (d) at a week 8 retest. Abbreviations: AS, Angelman syndrome mice; EE, enriched environment; SH, standard housing; WT, wild‐type littermates. Number of marbles ≥50% buried displayed as mean ± SEM. Two‐way analysis of variance (ANOVA) followed by Bonferroni‐corrected planned comparisons: *p < .05, **p < .01, ***p < .001

At 6 weeks postweaning, we also examined whether EE ameliorates the highly penetrant marble‐burying deficit seen in AS mice on the C57 background (Born et al., 2017; Huang et al., 2013; Sonzogni et al., 2019). Two‐way ANOVA analysis of data from male mice at 6 weeks postweaning revealed significant main effects of genotype (*p* < .0001) and housing (*p* = .0265) (Figure 3a). However, deficient marble burying in AS‐SH mice (vs. WT‐SH, *p* = .0005) was not significantly improved by EE (AS‐EE vs. AS‐SH, *p* = .1173). A follow‐up test 2 weeks later showed significant main effects of genotype (*p* < .0001) and housing (*p* = .0002) (Figure 3b). Deficient marble burying in AS‐SH mice (vs. WT‐SH, *p* < .0001) was completely rescued in AS‐EE mice (vs. AS‐SH, *p* = .0009; vs. WT‐SH, *p* = .2559).

Two‐way ANOVA analysis of data from female mice at 6 weeks postweaning revealed significant main effects of genotype (*p* < .0001) and housing (*p* = .0003) (Figure 3c). Deficient marble burying in AS‐SH mice (vs. WT‐SH, *p* < .0001) did improve with EE (AS‐EE vs. AS‐SH, *p* = .0024, AS‐EE vs. WT‐SH, *p* = .2430). However, a follow‐up test 2 weeks later showed that the improvement at 6 weeks had disappeared, revealing a significant main effect of genotype only (*p* < .0001) (Figure 3d). Indeed, we observed deficient marble burying mice in the AS‐SH mice (planned comparison vs. WT‐SH, *p* = .0003) that did not seem to be affected by EE (AS‐EE vs. AS‐SH, *p* = .8139; AS‐EE vs. WT‐SH, p = .0076). These differences in marble‐burying behavior between WT‐SH and both AS‐SH and AS‐EE mice seem to have been driven by an increase in marble‐burying behavior in the AS‐SH mice, so that it more closely resembles marble‐burying behavior in AS‐SH male mice than it did at the 6‐week timepoint, as well as increased marble burying in WT‐SH mice.

### 3.3 Open field

**FIGURE 4:**
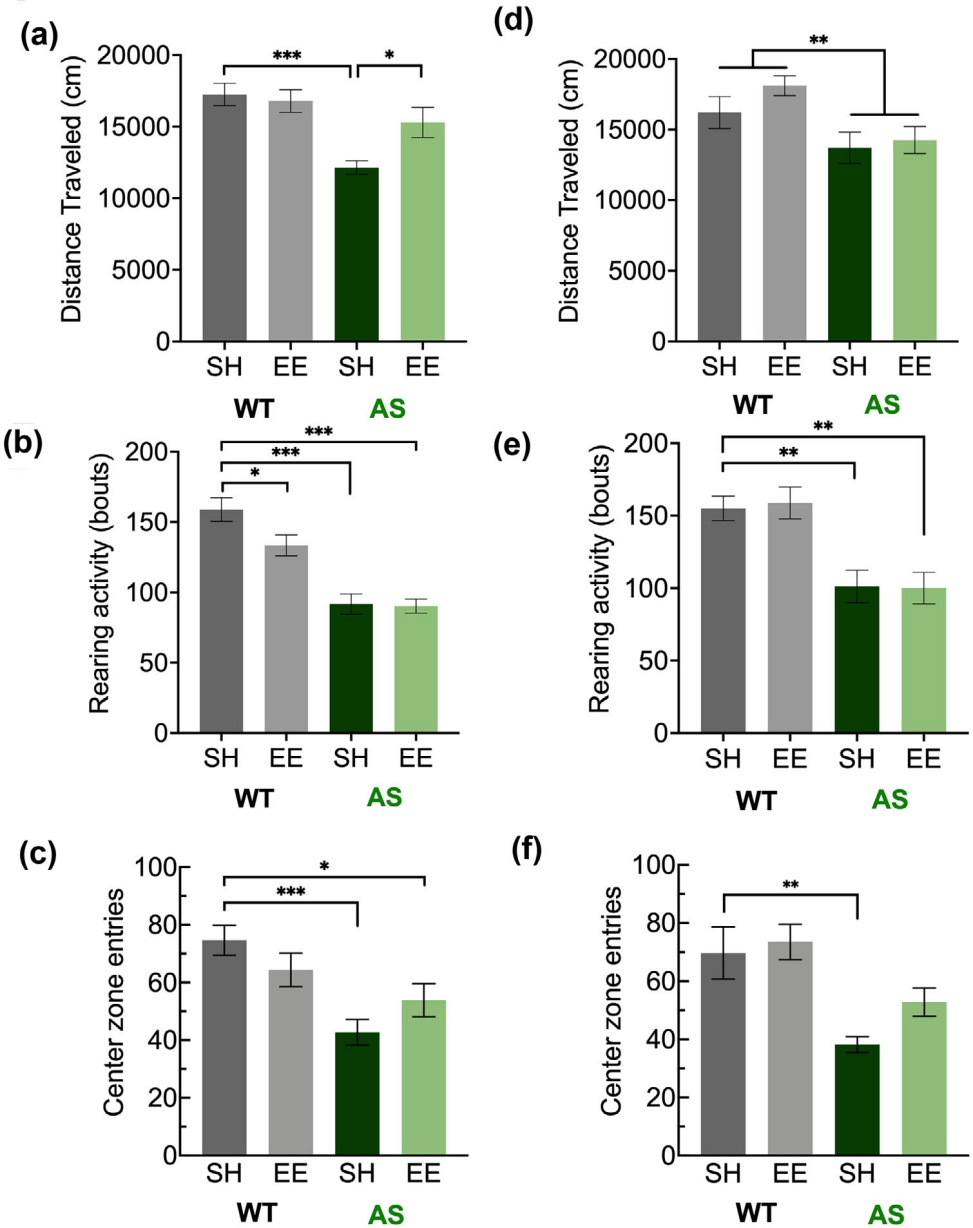
Environmental enrichment improves some aspects of exploratory activity in the open field in male AS mice after 7 weeks. Male AS mice in the enriched environment showed improvements in distance traveled (a), with no statistically significant improvement in deficient rearing behaviors (b) or thigmotaxis (c). Female AS mice in the enriched environment showed no statistically significant improvements in distance traveled (d), rearing behaviors (e), or thigmotaxis (f) relative to standard housing. Abbreviations: AS, Angelman syndrome mice; EE, enriched environment; SH, standard housing; WT, wild‐type littermates. All quantities displayed as mean ± SEM. Two‐way analysis of variance (ANOVA) followed by Bonferroni‐corrected planned comparisons: *p < .05, **p < .01, ***p < .001

Next, we examined whether 7 weeks of EE can improve behavioral deficits in the open field commonly seen in AS mice on the C57 background (Berg et al., 2021; Dutta & Crawley, 2020; Koyavski et al., 2019). Two‐way ANOVA analysis of distance traveled by male mice in the open field revealed a genotype x housing interaction (*p* = .0335) (Figure 4a). We observed a deficit in AS‐SH mice (vs. WT‐SH, *p* = .0001) that was improved in AS‐EE mice (vs. AS‐SH mice, *p* = .0340; vs. WT‐SH mice, *p* = .3132). There was no corresponding improvement in distance traveled due to housing in WT mice (*p* > .9999). When we examined the number of rearing movements in the same experiments, two‐way ANOVA analysis detected a significant main effect of genotype (*p* < .0001) with no significant main effect of housing (*p* = .0545) (Figure 4b). Indeed, we observed significant differences in AS rearing regardless of housing condition (AS‐SH vs. WT‐SH, *p* < .0001; AS‐EE vs. WT‐SH, *p* < .0001). When we examined the number of entries into the center zone, two‐way ANOVA analysis of data from male mice revealed a significant genotype x housing interaction (*p* = .0236) (Figure 4c). We observed that AS mice indeed made fewer center entries (vs. WT‐SH, *p* = .0001), with no significant improvement with EE (AS‐EE vs. AS‐SH, *p* = .1786, vs. WT‐SH, *p* = .0418). No improvements were seen in WT mice in EE (*p* = .2494).

In female mice, two‐way ANOVA analysis of distance traveled revealed a main effect of genotype (*p* = .0020) (Figure 4d). Planned comparisons revealed no significant group differences (*p* > .05), indicating mild deficits in distance traveled in AS mice. When we examined the number of rearing movements in the same experiments, two‐way ANOVA analysis of rearing events revealed a main effect of genotype (*p* < .0001) (Figure 4e). We confirmed a deficit in AS‐SH mice (vs. WT‐SH, *p* = .0037) that was not ameliorated in the presence of EE (vs. WT‐SH, *p* = .0057). When we examined the number of entries into the center zone, two‐way ANOVA analysis revealed a significant main effect of genotype (*p* = .0002) with no significant effect of housing (*p* = .0672) (Figure 4f). We observed that female AS mice were indeed deficient in center entries (vs. WT‐SH, *p* = .0024). Interestingly, center zone entries in AS‐EE mice were not significantly different from WT‐SH mice (*p* = .4590), suggesting improvement due to housing. However, because there were no corresponding significant differences between AS‐SH and AS‐EE mice (*p* = .1071), we could not conclude that this aspect of behavior had been rescued.

### 3.4 Rotarod

**FIGURE 5:**
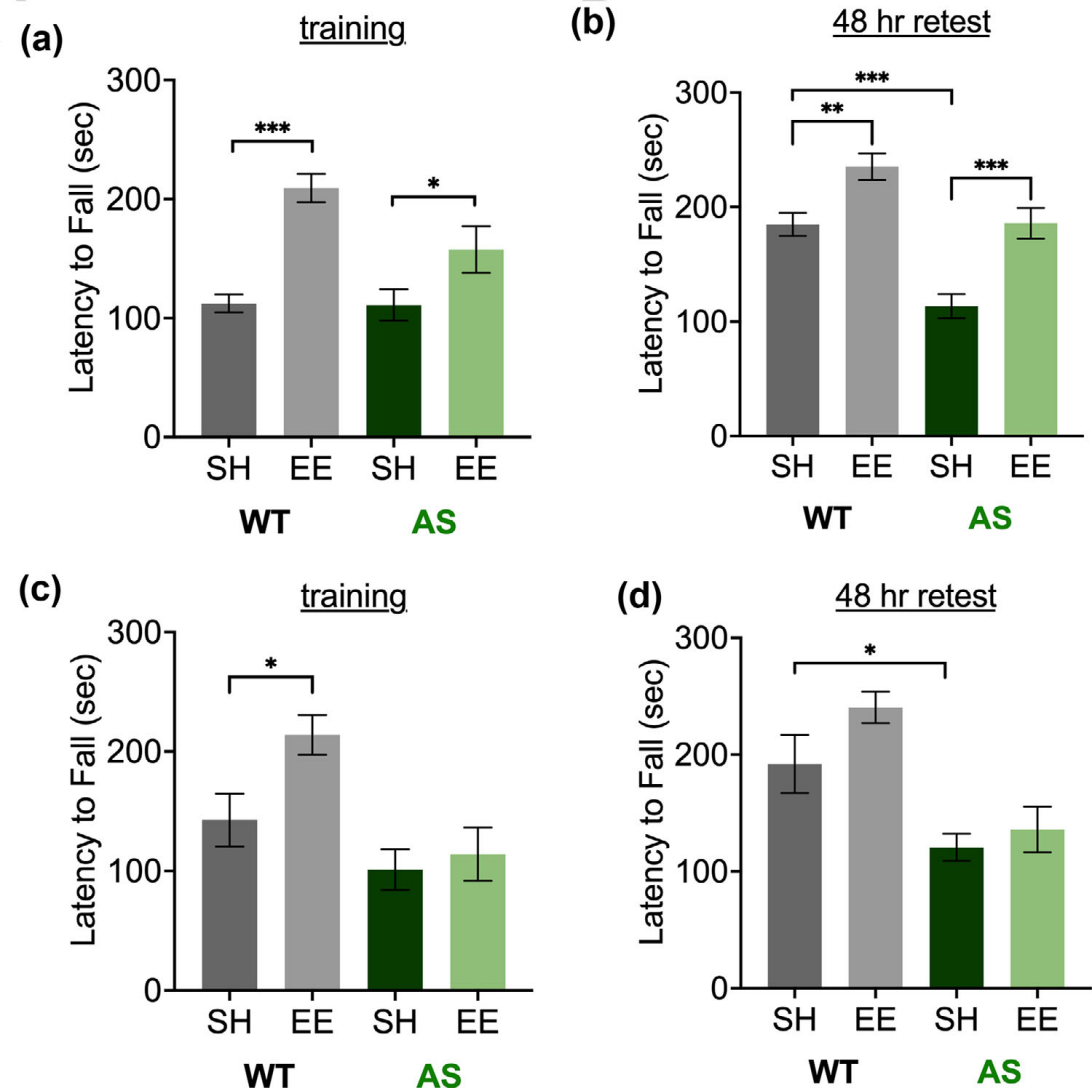
Environmental enrichment normalizes rotarod performance in male mice after 9 weeks. Improvement in the motor coordination deficit on the accelerating rotarod was observed in male AS mice (a and b) but not female mice (c and d) in environmental enrichment. Data show the training period (a and c) and a retest of motor learning 48 h later (b and d). Abbreviations: AS, Angelman syndrome mice; EE, enriched environment; SH, standard housing; WT, wild‐type littermates. Latency to fall, in seconds (maximum, 300 s), displayed as mean ± SEM. Two‐way analysis of variance (ANOVA) followed by Bonferroni‐corrected planned comparisons: *p < .05, **p < .01, ***p < .001

Then, we examined whether 9 weeks of EE rescues motor dysfunction on the accelerating rotarod seen in AS mice on the C57 background (Born et al., 2017; Huang et al., 2013; Jiang et al., 1998; Sonzogni et al., 2018). Two‐way ANOVA analysis of data from male mice revealed significant main effects of genotype (*p* = .0483) and housing (*p* < .0001) (Figure 5a). At this phase of the task, EE enhanced rotarod performance in both WT (*p* < .0001) and AS (*p* = .0467) mice, but notably there was no difference between WT‐SH and AS‐SH mice (*p* > .9999). During a retest of motor learning 48 h later, two‐way ANOVA analysis revealed significant main effects of genotype (*p* < .0001) and housing (*p* < .0001) (Figure 5b). At this phase of the task, we were able to replicate the rotarod deficit in AS‐SH mice (vs. WT‐SH, *p* = .0001), and we found that EE significantly normalized rotarod performance in AS‐EE mice (vs. AS‐SH, *p* = .0003; vs. WT‐SH, *p* > .9999) and improved rotarod performance in WT‐EE mice (vs. WT‐SH, *p* = .0029).

The same experiments in female mice revealed no rescue of the rotarod phenotype. Two‐way ANOVA analysis revealed a significant main effect of genotype (*p* = .0008) and housing (*p* = .0374) (Figure 5c). However, we observed a significant difference in rotarod performance only in WT (planned comparison, WT‐SH vs. WT‐EE, *p* = .0186). During a retest of motor learning 48 h later, two‐way ANOVA analysis revealed a significant main effect of genotype (*p* < .0001) (Figure 5d). We confirmed the documented deficits in AS‐SH mice (vs. WT‐SH, *p* = .0161), but found no improvements in performance due to EE (vs. AS‐SH, *p* > .9999).

### 3.5 Forced swim

**FIGURE 6:**
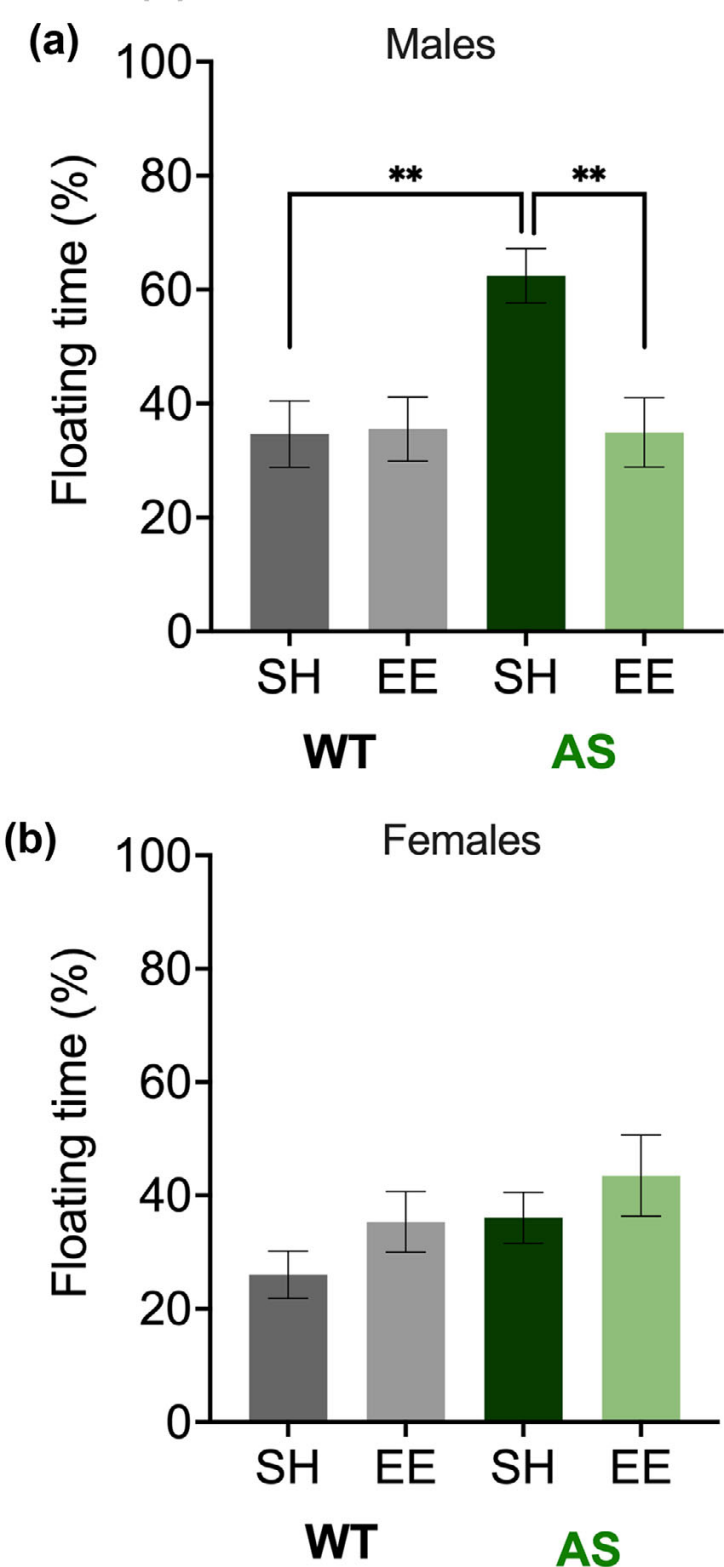
Environmental enrichment corrects forced swim performance in male mice after 11 weeks. The forced swim deficit in male AS mice improved with environmental enrichment (a), but similar changes were not seen in female mice (b). Abbreviations: AS, Angelman syndrome mice; EE, enriched environment; SH, standard housing; WT, wild‐type littermates. Floating time, as a percentage of 4 min, displayed as mean ± SEM. Two‐way analysis of variance (ANOVA) followed by Bonferroni‐corrected planned comparisons: *p < .05, **p < .01, ***p < .001

Finally, we tested whether EE rescues forced swim deficits that have been documented in AS mice (Silva‐Santos et al., 2015; Sonzogni et al., 2018). Two‐way ANOVA analysis of the data from the male mice revealed a significant genotype x housing interaction (*p* = .0198) (Figure 6a). We found a difference between mice in SH (WT‐SH vs. AS‐SH, *p* = .0015) that was ameliorated by EE (AS‐SH vs. AS‐EE, *p* = .0076; WT‐SH vs. AS‐EE, *p* > .9999). There was no impact of EE on WT mice (WT‐SH vs. WT‐EE, *p* > .9999). Two‐way ANOVA analysis of the data from female mice revealed neither significant main effects nor a significant interaction (*p* > .05) (Figure 6b).

## 4 DISCUSSION

In the present study, we set out to determine the degree to which enrichment of the environment rescued behavioral phenotypes associated with AS in mice. We tested the effects of 6–12 weeks of postweaning sensory, cognitive, and physical enrichment on a battery of behavioral tasks that have been shown to be highly reproducible across laboratories and have face validity with AS in patients (Rotaru et al., 2020; Sonzogni et al., 2018). We recapitulated the behavioral deficits in both male and female AS mice but found that our EE protocol was far more successful at normalizing these phenotypes in male mice than in female mice. These findings suggest that EE may have therapeutic value in AS, but sex differences and other aspects of enrichment merit further consideration.

Our EE protocol was remarkably effective in male AS mice. First, we observed a complete rescue of rotarod performance at the 48‐h retest after 9 weeks. Second, we observed a complete rescue of horizontal exploratory behavior in the open field to WT levels after 7 weeks. Third, we observed a complete normalization of marble burying to WT levels after 6 weeks. Fourth, we observed a rescue of immobility in the forced swim task. Finally, we observed a correction of weight. These findings are consistent with a body of evidence showing that EE is effective at correcting some phenotypes in mouse models of neurodevelopmental disorders. In mouse models of autism—which is comorbid in most AS patients (Mertz et al., 2014)—EE has been demonstrated to correct hypoactivity in the open field (Queen et al., 2020; Suemaru et al., 2018; Yamaguchi et al., 2017) or normalize ethologically relevant repetitive behaviors like marble burying (Mansouri et al., 2021; Reynolds et al., 2013). Interestingly, these studies showed a decrease in marble burying to WT levels following EE, rather than the increase we observed here. In mouse models of Rett syndrome—a disorder that resembles AS in its developmental delay, absent speech, ataxia, and seizure susceptibility but is most distinct in its regressive phenotype (Jedele, 2007; Tan et al., 2014)—EE has been demonstrated to restore motor coordination deficits on the rotarod (Kondo et al., 2008; Lonetti et al., 2010) and correct hypoactivity in the open field (Nag et al., 2010). In mouse models of Fragile X syndrome—which shares autism incidence, sensory sensitivity, and intellectual disability with AS (Heald et al., 2020)—EE has been shown to correct hyperactivity in the open field (Restivo et al., 2005). However, none of these studies demonstrated the same high degree of correction across the same suite of behaviors examined in the current study. Thus, this combination of EE parameters seems to be potent in male AS mice.

Interestingly, 7 weeks of EE in male AS mice did not rescue rearing behavior and center zone entries in the open field, behaviors that have been used to probe anxiety in mice (Kulesskaya & Voikar, 2014; Sturman et al., 2018), including in AS mouse models (Born et al., 2017; Godavarthi et al., 2012; Guzzetti et al., 2018; Koyavski et al., 2019; Sonzogni et al., 2018). These results contrast with some prior studies described above that see improvements in anxiety‐related behavior with EE (Lacaria et al., 2012; Lonetti et al., 2010; Queen et al., 2020; Restivo et al., 2005). However, the finding of an uncorrected rearing deficit may not be explained by unmitigated anxiety; rather, it is possible that AS mice have muscle weakness that that is not ameliorated with 7 weeks of EE, despite restored horizontal movement in the open field and improvement on the rotarod after 9 weeks of EE. Indeed, recent studies have started to document subtle, clinically relevant motor deficits in AS rodent models (Berg et al., 2021, 2020; Petkova et al., 2021). Nevertheless, anxiety is a key part of the clinical presentation of AS (Wheeler et al., 2019). Therefore, future work should examine the effect of short‐term EE on highly reproducible tasks that more clearly dissociate anxiety‐related behaviors from confounding motor deficits in AS mice, including the elevated‐plus maze (Born et al., 2017; Ciarlone et al., 2016, 2017; Dutta & Crawley, 2020), the light‐dark task (Dutta & Crawley, 2020; Godavarthi et al., 2012; Jiang et al., 2010), or other tasks (Crawley, 2007). One prior study indicates that long‐term EE rescues behavior in the light‐dark task in male AS mice (Jamal et al., 2017), so it will be fruitful to examine the role of EE on this behavior on a shorter timescale in both sexes. It would also be beneficial for future work to examine more closely the effect of EE on motor behavior in AS, including clinically relevant phenotypes like gait (Berg et al., 2021).

It should be noted that we did not necessarily expect our protocol to rescue as many of the behaviors as we tested. For example, in mouse models of autism, the behaviors for which EE is documented as either being highly effective or largely ineffective can vary from model to model, as in the studies described above. There is even one example in which EE failed to rescue any of the disease‐related phenotypes examined in a mouse model of autism (Hulbert et al., 2018). The effectiveness of EE for the suite of behaviors here—or the ineffectiveness of EE on behavior elsewhere—may be due to the interaction between the environment and disease pathophysiology at a particular point in time and may depend on the type and duration of enrichment (Kondo & Hannan, 2019; Nithianantharajah & Hannan, 2006). Moreover, among studies that have tested novel therapeutic approaches in AS model mice, some of the highly reproducible behavioral phenotypes are more frequently rescued than others. Across 16 recent studies that were able to rescue at least one of the behaviors tested here, motor coordination deficits on the rotarod were most commonly corrected, with the phenotype completely or partially rescued in over half of the studies (nine of 16: Adhikari et al., 2021; Cruz et al., 2021; Guzzetti et al., 2018; Judson et al., 2021; Kumar et al., 2019; Schmid et al., 2021; Silva‐Santos et al., 2015; Van Woerden et al., 2007; Wolter et al., 2020) and no rescue in the rest of the studies (Berg et al., 2020; Gu et al., 2019; Hethorn et al., 2015; Milazzo et al., 2021; Schultz & Crawley, 2020; Sonzogni et al., 2018; Wang et al., 2019). Partial or complete rescue occurred least frequently in open field thigmotaxis (one of four studies in which it was examined: Guzzetti et al., 2018) and marble burying (four of 10 studies in which it was examined: Cruz et al., 2021; Judson et al., 2021; Schmid et al., 2021; Silva‐Santos et al., 2015). Together, this body of evidence suggests that some disease‐related phenotypes may be more easily rescued than others (though it should be noted that factors including genetic background and age of intervention may have influenced the effectiveness of any given approach). Further research should probe the mechanistic basis that underlies the ability of the combination of EE parameters in this study to shape a wide range of AS‐relevant behaviors.

In stark contrast to the success of EE in male AS mice, our EE protocol was substantially less effective in female AS mice. Here, only the previously documented weight phenotype (Ciarlone et al., 2016; Huang et al., 2013) showed improvement. Although previous work showed that there are no sex differences in the behavioral tasks we selected in AS‐SH mice (Born et al., 2017; Koyavski et al., 2019; Sonzogni et al., 2018), one recent study (Koyavski et al., 2019) showed that there were sex differences in odor perception and object exploration and hinted that other sex differences may be uncovered as the literature on female AS mice expands. Indeed, sex differences in the effectiveness of EE are not unheard of. In some studies of EE in rodents, EE was beneficial for males but not females (Doreste‐Mendez et al., 2019; Kentner et al., 2018; Queen et al., 2020; Stam et al., 2008; Wood et al., 2010), while in other studies, EE was beneficial in females but not males (Lin et al., 2011; Martínez‐Cué et al., 2002; Stam et al., 2008). How might these differences arise? Previous studies on EE in mice of both sexes have documented differences in how EE interacts with hormonal state and stress response to anxiogenic tasks (Girbovan & Plamondon, 2013; Kentner et al., 2018). Other studies suggest that EE in female mice may not engage the same molecular mechanisms to the same degree as in male mice (Chourbaji et al., 2012; Kentner et al., 2018; Lin et al., 2011; Queen et al., 2020). If the effect of EE on behavioral phenotypes in AS mice depends on brain‐deried neurotrophic factor upregulation and glucocorticoid receptor downregulation, as has been reported previously (Jamal et al., 2017), it is possible that female mice do not respond in the same way because of the failure of EE to engage these molecular mechanisms. Furthermore, the most effective EE parameters may not be identical for male and female AS mice. In other studies of EE, female mice tend to benefit more from some forms of enrichment, like social enrichment, than others (Chourbaji et al., 2008; Kondo et al., 2016; Lambert et al., 2005; Pietropaolo et al., 2004). It is also possible that other behavioral differences in AS mice, including recently detected sex differences in odor perception and object exploration (Koyavski et al., 2019), may account for fundamental differences in how male and female mice engage with the enriched environment. In the same vein, it is possible that female AS mice require longer bouts of EE or need to begin EE at a different developmental time point. It will be interesting to examine how altering enrichment parameters might make this intervention more effective for female AS mice.

Our findings are distinct from the results of the only other existing study of EE in AS mice (Jamal et al., 2017) in a few important ways. First, although both studies show that postweaning enrichment is sufficient to rescue deficits on the rotarod, we were able to observe statistically significant differences in AS mouse behavior after just 9 weeks rather than 16–32 weeks. The researchers reported a lack of a significant difference after 8 weeks, but this finding may be an indication that the experiment was underpowered or that there were important differences in EE protocol. Second, we examined behaviors that cover a larger swath of disease‐related phenotypes and are robust and highly reproduced (Sonzogni et al., 2018). Our study adds a robust battery of exploratory, anxiety‐related, and species‐typical repetitive behaviors to the previous findings. These tasks provide relatively quick endpoint assays for the effectiveness of EE, or drugs that engage the same mechanisms as EE (Kondo & Hannan, 2019; McOmish & Hannan, 2007; Solinas et al., 2021). Third, our study used both male and female mice, rather than just male mice, to document whether sex is a variable in the effectiveness of EE. In total, our findings step forward in understanding enrichment as an intervention for AS.

Our study also has important limitations. The current study was limited to *Ube3a^tm1Alb^* mice (Jiang et al., 1998) on the C57BL6/J background, and EE was administered for 6–12 weeks immediately upon weaning. Differences in behavior due to background strain are well‐documented in the literature (Born et al., 2017; Huang et al., 2013; Sonzogni et al., 2018), and it is possible that our EE protocol will not be as effective on other background strains, despite our focus on highly reproducible behaviors across backgrounds. It is also possible that mouse models carrying mutations relevant to AS in human patients (Jiang et al., 1998; Rotaru et al., 2020) will respond differently to this EE protocol. In addition, it will also be useful to understand how the type and duration of EE, the time of onset of EE, and the removal of long‐term EE affect the effectiveness of the intervention. Future work addressing these areas will help us determine the broad applicability of EE to AS.

Future work should also examine other highly penetrant phenotypes associated with AS and examine the role EE has on the pathophysiology of the disease in rodents. Seizure susceptibility is one of the commonly documented features of AS in both human patients and model mice. Previous research in other mouse models indicates that EE is effective at reducing epilepsy (Morelli et al., 2014; Young et al., 1999), and it is reasonable to hypothesize that EE is also effective at reducing seizures in AS mice. It would also be useful to know how effective EE is at correcting phenotypes that echo the cognitive symptoms of AS in patients. Although deficits in cognitive tasks like the novel object recognition task, fear conditioning, and the Morris water maze have been reported, they are relatively modest (Sonzogni et al., 2018). Nevertheless, work on EE in mouse models of autism, Fragile X syndrome, and Rett syndrome shows substantial rescue of cognitive behavior (Jamal et al., 2017; Lacaria et al., 2012; Lonetti et al., 2010; Restivo et al., 2005). Finally, future research should attempt to document other forms of EE‐mediated recovery in AS mice at the cellular and systems levels. Understanding these mechanisms and the conditions under which they are engaged will increase the importance of our findings.

The translatability of these results to AS patients remains to be seen. A few studies with autism patients suggest that long‐term sensorimotor stimulation can lead to significant correction of cognitive disruption and behavioral dysfunction (Woo & Leon, 2013; Woo et al., 2015). These clinical findings show that some form of enrichment—or therapies that engage the same mechanisms as enrichment (Hill‐Yardin & Hannan, 2013)—may be effective in AS patients, given the results of the current study. To our knowledge, no such study currently exists. In addition, the finding of sex differences in response to environmental enrichment suggests that slightly different therapeutic approaches may need to be taken with males and females undergoing treatment for AS. Any future study of enrichment in patients will need to carefully account for sex, type of stimulation, and desired outcome.

### PEER REVIEW

The peer review history for this article is available at https://publons.com/publon/10.1002/brb3.2468

## Supporting information

Supporting Information. Spreadsheet containing results from statistical tests presented in this study. Each set of results is displayed on separate tabs by corresponding figure number. Transformed data used where reported. Statistical significance for main effects and interactions in the two‐way ANOVA (columns F‐G) and Bonferroni‐corrected planned comparisons (columns I‐J): *p < .05, **p < .01, ***p < .001. XLS format.

## Data Availability

Data that support the findings of this study and custom code used to analyze data are available from the corresponding author upon reasonable request.
